# Effectiveness of Chitosan against Mature Biofilms Formed by Food Related Bacteria

**DOI:** 10.3390/ijms12010817

**Published:** 2011-01-21

**Authors:** Belen Orgaz, Maria M. Lobete, Carmen H. Puga, Carmen San Jose

**Affiliations:** Department of Nutrition, Food Science and Technology, Faculty of Veterinary, University Complutense of Madrid, Spain; E-Mails: maria.mlobete@gmail.com (M.M.L.); chpuga@estumail.es (C.H.P.); serran@vet.ucm.es (C.S.)

**Keywords:** biofilm, chitosan, pronase, disinfection, food industry, Listeria monocytogenes, Bacillus cereus

## Abstract

Chitosan has proven antimicrobial properties against planktonic cell growth. Little is known, however, about its effects on already established biofilms. Oriented for application in food industry disinfection, the effectiveness of both medium molecular weight (MMW) chitosan and its enzymatically hydrolyzed product was tested against mature biofilms of four pathogenic strains, *Listeria monocytogenes*, *Bacillus cereus*, *Staphylococcus aureus* and *Salmonella enterica*, and a food spoilage species, *Pseudomonas fluorescens*. Unexpectedly, log reductions were in some cases higher for biofilm than for planktonic cells. One hour exposure to MMW chitosan (1% w/v) caused a 6 log viable cell reduction on *L. monocytogenes* monospecies mature biofilms and reduced significantly (3–5 log reductions) the attached population of the other organisms tested, except *S. aureus*. Pronase-treated chitosan was more effective than MMW chitosan on all tested microorganisms, also with the exception of *S. aureus*, offering best results (8 log units) against the attached cells of *B. cereus*. These treatments open a new possibility to fight against mature biofilms in the food industry.

## 1. Introduction

In most environments the majority of microorganisms are able to grow as biofilms [1], where they express a different phenotype from their planktonic counterparts [2]. The main feature of this phenotype is the production of extracellular materials that build an adhesive gel, the matrix, embedding the cells and protecting them from shear forces and harsh conditions, including presence of most antimicrobial agents [3]. Food contact surfaces are good substrata for biofilm development. Although strict cleaning and disinfection procedures can generally assure suitable hygienic conditions in the food industry, destroying planktonic cells and biofilms starting to be formed, they may fall short for the elimination of biofilms that are already well developed. These tend to settle on sites that are especially difficult to clean, due to difficult access, surface irregularities or retention of sticky raw materials. Microbial cell transfer from biofilms to foods, particularly after their hygienization, is a hazard for food safety and quality [4,5].

Chitosan, a polysaccharide industrially derived from partial deacetylation of chitin, is an antimicrobial that has shown promise, in solution or as surface coating [6,7]. The mechanism of action has been ascribed mostly to its primary amino groups that could be involved in electrostatic interaction with anionic cell wall components, thus leading to changes in membrane permeability [8] and even disruption of its barrier properties [9]. One of the major obstacles for chitosan use is poor solubility in water [10]. Thus, numerous studies have aimed to obtain water-soluble chitosan and chitooligosaccharides (COS) using different methodologies, such as reduction processes [11], chemical modification [12], chemical or enzymatic hydrolysis [7,9,13–15]. Interestingly enough, chitosan seems to be a good substrate for a wide spectrum of enzymes, including proteases and carbohydrate-degrading enzymes [16]. The resulting COS are in some cases more effective antimicrobials than native chitosan [13,15]. In addition, antimicrobial synergy could exist between COS and chitosan-degrading enzymes, such as Pronase [17]. The smaller molecular weight (MW) facilitates the molecule’s access into the cells where they can even bind to DNA [8,18]. Factors such as the exposed microorganism, time of exposure and growth phase at that time, have been reported to influence the extent of the antimicrobial action of chitosan and COS [19,20].

Most of the published work on bactericidal effects of chitosan and its derivate compounds are studies against planktonic microorganisms [13,21,22] or intended for inhibition of bacterial attachment [23–25]. Much less is known, however, about the behavior of chitosan and its derivates against well established biofilms [26], which are assumed to be more recalcitrant to cleaning and disinfection practices than planktonic or newly attached cells [27]. Impaired diffusion rates and other aspects of biofilm lifestyle, such as slower division rates, are thought to contribute to the generally recognized higher resistance of biofilms to antimicrobials [28]. Our purpose in the present study was to evaluate the activity of different concentrations of native and degraded chitosan against mature biofilms of four strains of bacterial species frequently associated with foodborne diseases: *Listeria monocytogenes*, *Bacillus cereus*, *Staphylococcus aureus* and *Salmonella enterica* were selected, plus a strain of *Pseudomonas fluorescens,* well known for its rapid formation of thick biofilms and high spoilage potential in refrigerated dairy products. A set of *L. monocytogenes* food industry isolates were also tested for in-strain vulnerability comparison. Enzymatically degraded chitosan was here achieved using Pronase^®^, a natural mixture of extracellular, compatible and complementary proteolytic enzymes, produced by some strains of *Streptomyces griseus* [29].

## 2. Results and Discussion

**Figure 1:**
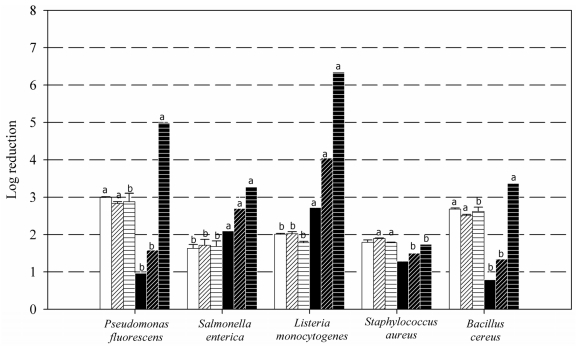
Log reduction of planktonic (white bars) and biofilm (black bars) cells of five bacterial strains, after 60 min exposure to 0.01% (plain bars), 0.1% (slanted line bars) and 1% (horizontal line bars) chitosan. Statistical comparison was made between planktonic and biofilms log reduction values exposed to the same chitosan concentration. Values showing a different superscript letter were significantly different (p < 0.05) (n = 6).

Both planktonic cells and mature biofilms (with 10^7^–10^9^ CFU cm^−2^) of the five bacterial species were used as targets for three concentrations, 0.01, 0.1 and 1%, of medium MW chitosan (Figure 1). Concerning planktonic cells, a maximal log reduction of about 3 was observed for both *Pseudomonas* and *Bacillus*, and no more than 2 for the other three species. Practically no effect of chitosan concentration, within this interval, was observed on planktonic cell log reduction. The effect of concentration upon cell log reduction was much more intense on biofilms. Maximum effectiveness (≥6 log units) with 1% chitosan was registered on *Listeria* biofilms, which had an initial population level of 2 × 10^6^ CFU cm^−2^. Cells in *Pseudomonas* biofilms, initially 2 × 10^9^ CFU cm^−2^, underwent a 5 log reduction effect. Those of *Salmonella* (initially 2 × 10^6^ CFU cm^−2^) and *Bacillus* (6 × 10^8^ CFU cm^−2^) showed about 3 log cell reductions. The lowest effect was obtained on *Staphylococcus* biofilms (initially 9 × 10^6^ CFU cm^−2^), less than 2 log reduction. Treatments with 0.1% solutions still had a considerable impact on *Listeria* biofilms (4 log reduction) and a moderate impact on those of *Salmonella* (2.5 log), but only a 1.5 log reduction on biofilms of the other three tested organisms. When chitosan was lowered to 0.01%, still 2.5 and 2.0 log reductions were achieved on counts of *Listeria* and *Salmonella,* respectively, however viability losses of the other three organisms were only about 1 log unit.

The differences in response to chitosan shown in Figure 1 do not support the contention of a role for cell wall Gram type. Peculiarities of the type of matrix could perhaps better explain the widely diverse results. The typical sparse matrix of the single-species *L. monocytogenes* biofilms [30,31] could be a reason for their higher vulnerability, though *Pseudomonas* biofilms, which are known to have a thick matrix, are also highly susceptible. Though data on gross chemical composition of certain biofilms and some of their exopolysaccharides are available [3,32,33], information on the physical and physicochemical properties of the biofilm matrices of different organisms, such as mess size and elasticity, amount and distribution of charges or polymeric junctions, or other properties affecting diffusion rates and retention abilities, is unfortunately very scarce [3]. Some exopolysaccharides such as those of *Pseudomonas* are known to be polianionic [32,34] whereas others such as the adhesins of *Staphylococcus* are policationic [3,35]; this could be related to the response of their respective biofilms to chitosan exposure.

It is generally accepted that biofilm-embedded cells enjoy a protection against multiple stress conditions, such as lack of water or nutrients, or presence of antimicrobial agents [36]. Chitosan, however, seemed here to be more damaging against sessile cells (except *S. aureus*) than against planktonic cells, at least with the highest (1%) concentration used. Chavant *et al*. (2004) have reported on alkaline agents having a similar effect on biofilm and planktonic cells of *L. monocytogenes* [37]. Cabo *et al*. (2009), using *S. aureus* biofilms, reported higher effectiveness of benzalkonium chloride against attached cells than on planktonic ones [38]. Penetration of chitosan network into the matrix could possibly depend on its charge and thickness and also on chitosan’s size, deacetylation degree and concentration. It is possible that, at low chitosan concentrations, uronic and other acid residues of anionic exopolysaccharides such as those present in the *Pseudomonas* matrix, bind the amino groups of chitosan acting as traps, thus protecting the embedded cells from the biocide. When those traps get saturated, additional chitosan molecules might, at least in 60 min, access the embedded cells and accumulate in their vicinity, causing an “intensification” effect not attainable on planktonic cells. Further research is needed to elucidate the mechanism underlying the results of Figure 1 for *P. fluorescens*.

**Figure 2:**
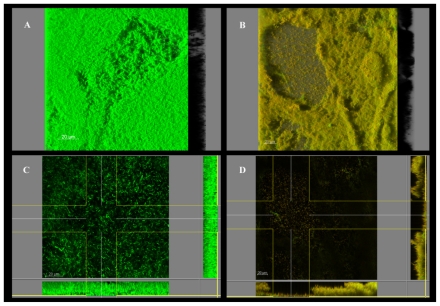
Confocal Scanning Laser Microscopy (CLSM) images of *P. fluorescens* biofilms before and after 60 min immersion in 1% chitosan. 3-D view before (**A**) and after (**B**) treatment. Extended section view (z-y and z-x planes) before (**C**) and after (**D**) treatment; these images correspond to the deepest layers of the biofilm. Color allocation: green = live cells (Syto9); red = dead cells (propidium iodide). Scale bar = 20 μm.

Microscopy images of control and treated biofilms of different bacteria may help to understand the different response patterns. Figure 2 shows Confocal Scanning Laser Microscopy (CLSM) images dyed with Live-Dead indicator system, from *P. fluorescens* biofilms before and after 1% chitosan treatment. Apart from a clear change of color from green to red, indicating extensive cell permeabilization and death, chitosan appeared to cause morphological changes. From a rather smooth, thick and homogeneous biofilm structure in the control, a thinner and more irregular landscape was generated after treatment, indicating substantial cell and/or microcolony detachment. Whether this is a case of cell death inducing collapse of the matrix integrity, or the opposite, matrix alteration allowing chitosan penetration to reach and kill the embedded cells, it is hard to know at this stage.

**Figure 3:**
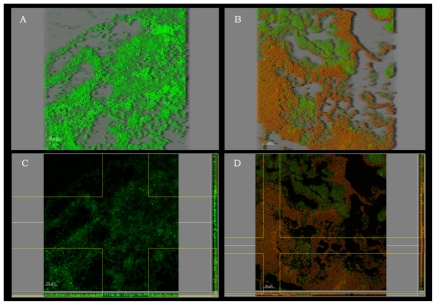
CLSM images of *S. aureus* biofilms before and after 60 min immersion in 1% chitosan. 3-D view before (**A**) and after (**B**) treatment. Extended section view (z-y and z-x planes) before (**C**) and after (**D**) treatment; these images correspond to the deepest layers of the biofilm. Color allocation: green = live cells (Syto9); red = dead cells (propidium iodide). Scale bar =20 μm.

Poor results of chitosan against *S. aureus* may be the consequence of specific features of their biofilm matrix, cell wall or membrane, and/or some intracellular events. Figure 3 displays *S. aureus* native biofilm structure showing a hollow space, porous matrix appearance, similar to that observed by Bridier *et al*. (2010) [31], which looks favorable to penetration. No morphological changes in *S. aureus* biofilm structure were apparently caused by chitosan exposure. Carlson *et al*. (2008), however, achieved a 5.5 log reduction in the attachment of *Staphylococcus epidermidis* when coating packaging material surfaces with highly deacetylated medium MW chitosan [23]. Raafat *et al*. (2008) studied the response to chitosan of *Staphylococcus simulans*, suggesting changes in the expression of a stress and autolysis regulation mechanism, apart from alterations of the cell wall [39].

As chitosan quantitative effects were highest on *L. monocytogenes* Scott A, we checked whether a similar effect was also obtained on the biofilms of five other strains of the same species (initially about 2 × 10^7^ CFU cm^−2^). A concentration of 0.1% was selected to measure potential differences in susceptibility among strains (Table 1). *L. monocytogenes* Scott A registered the highest log reduction of the six strains. These data give a good prospect for the use of chitosan in disinfection, in particular for periodic or sporadically control of *L. monocytogenes* in food processing or handling facilities [40].

**Figure 4:**
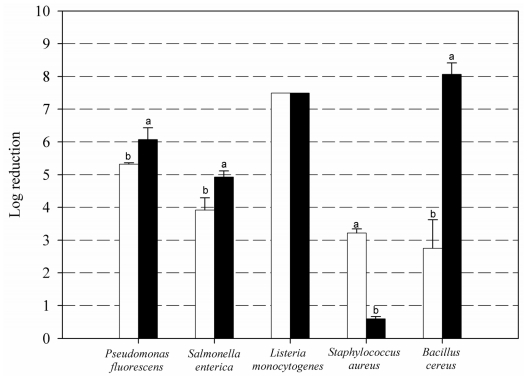
Log reduction of biofilm cells of various microorganisms, after 60 min immersion in 1% chitosan (white) and 1% Pronase^®^-hydrolyzed chitosan (black) (n = 6).

Figure 4 compares the effects of the 1% chitosan and Pronase^®^ hydrolyzed chitosan (PHC) treatments on the same species as in Figure 1. Again, it is hard to say whether the very variable responses to the PHC treatments depend on features pertaining to the cells themselves or their respective biofilm structures.

The most outstanding result of Figure 4 result was the vast effect of PHC on *B. cereus* biofilms (8 log units). These results can be related to those previously described with planktonic cells of the same species [13]; these authors achieved a higher growth inhibitory activity towards both *B. cereus* and *Escherichia coli* with a chito-oligomeric-monomeric mixture prepared with papain and Pronase, than with native chitosan.

The poorest results with PHC were observed for *S. aureus* biofilms, whose cells were almost undamaged (under 1 log reduction). Fernandes *et al*. (2008) [22] and Eaton *et al*. (2008) [41] also observed that high and medium MW chitosans were more effective than lower MW derivatives towards planktonic *S. aureus*.

There has been attempts to establish a relationship between the Gram character and the size of the most effective chitosan against planktonic cells. Wang *et al*. (2008) [9], employing bromelain chitosan hydrolysates, failed to observe any relationship with the Gram+/− character. Other authors claim that native chitosan has a stronger action against Gram positives whereas chitooligomers are more effective against Gram negatives [22,42]. Kumar *et al*. (2007) [15], however, achieved complete growth inhibition of *B. cereus* with 0.01% of low MW chitosan within 1 h, needing up to 5 h to reach a comparable effect on *E. coli*.

Many enzymes unintentionally present in food raw materials could have the effect of degrading chitosan, thus making it more effective against certain microorganisms. A combined system using chitosan, COS and several enzymes has been previously developed for biofilm removal [17]. It consisted of capsules for temporal confinement of Pronase with a chitosan and alginate shell, which had to be immersed in a carbohydrase solution to initiate biofilm attack. The carbohydrase would break the capsule shell after some time, allowing the proteinase mixture to come out, both producing COS and degrading the biofilm matrix, previously eroded by the soluble carbohydrase. Pronase would then eventually destroy the soluble enzyme, the previously embedded biofilm cells and its own activity. This process integrates matrix removal and cell killing and has the advantages of a closed process whose effluents, not carrying residual enzymatic activities, would not damage the well balanced biological treatments of wastewater stations.

Chitosan’s main food-related use at present is as a weight-loss food supplement, whose production amounted to about 50% of the commercial product in the year 2000 [7]. The EU considers chitosan as one of the “miscellaneous bioactive substances” that can be included in food supplements (in the same group as lycopene and soy isoflavones, for instance). Besides, certain traditional foods, such as mushrooms, have chitosan as a natural component [43]. The estimated intake of chitosan residues that could get incorporated into foods as a result of one of its antimicrobial uses, from packaging materials or residues left by food equipment disinfection products, will presumably be far below the already tolerated dietary intake.

## 3. Experimental Section

### 3.1. Bacterial Strains

Six strains of *Listeria monocytogenes*, reference strain Scott A (serovar 4b), the following four isolated from poultry: H66a (serovar 1/2a), H66b (serovar 1/2c), H63a (serovar 1/2b), CAL17a (serovar 4b) and F6861 (a cheese strain), *Salmonella enterica* serovar Enteritidis ATCC 13076, *Staphylococcus aureus* CECT 4013, *Pseudomonas fluorescens* ATCC 948^TM^ and *Bacillus cereus* UN2814, were selected as a biofilm former organisms. All strains were stored at −20 °C in Tryptone Soya Broth (TSB) (Oxoid) with 15% glycerol. Preinocula were obtained under shaking (80 rpm) at 37 °C/24 h in the case of *Bacillus, Listeria*, *Staphylococcus* and *Salmonella*, and at 21 °C/24 h for *Pseudomonas*, all in TSB and reaching up to mid exponential phase. Cells were harvested by centrifugation at 4000 × g for 10 min, washed twice in sterile TSB and their OD^600^ adjusted in order to reach 10^4^ CFU mL^−1^ in cultures after inoculation.

### 3.2. Experimental Systems

Biofilms were developed on single-use 22 × 22 mm thin, borosilicate commercial microscope glass coverslips as described in [44]: 16 coverslips were held vertically by marginal insertion into the narrow radial slits of a Teflon carousel platform (6.6 cm diameter). The platform and its lid were assembled by an axial metallic rod for handling. Coverslips, carousel and the covered 600 mL beaker were heat-sterilized as a unit, before aseptically introducing 60 mL of inoculated TSB. Incubation was carried out at 21 °C in the case of *P. fluorescens* and at 37 °C for the rest of the microorganisms, in a rotating shaker at 80 rpm. Under these conditions, biofilm growth ocupied about 70% of the coverslip’s surface. To obtain mature biofilms with an attached cell density of 10^6^–10^9^ CFU cm^−2^, an incubation of 48 h at 21 °C was needed for *Pseudomonas* cultures, and 72 h at 37 °C for the other organisms.

For planktonic cell cultivation, Erlenmeyer flasks containing 50 mL of TSB were inoculated with the same cell density and kept under the same incubation conditions used for biofilm development.

### 3.3. Chitosan Preparations and Treatments

Chitosan was purchased from Sigma Aldrich, having 75–85% deacetylation degree and medium molecular weight, ranging from 190,000 to 300,000 Da. For biofilm assays, a 1% (w/v) chitosan solution was prepared in 1% (v/v) acetic acid. From this solution, 0.1% chitosan and 0.01% chitosan were prepared in distilled water. A 1% (w/v) chitosan solution was treated with Pronase^®^ (Roche Molecular) (1 mg/mL) at 25 °C for 1 h. Proteolytic activity was inactivated by heating for 10 min at 100 °C.

For mature biofilm treatment, the coverslips were aseptically extracted from the carousel platform with sterile tweezers and dipped for 1 min in sterile saline (0.9% w/v), in order to eliminate weakly attached cells. Then they were individually immersed into Falcon test tubes containing 15 mL of the corresponding cleaning solution, for 1 h at 20 °C. After treatment, the washing step with saline was repeated before cell recovery and counting.

For the planktonic assays, a 2% (w/v) chitosan solution in 1% (v/v) acetic acid was prepared, diluting it with distilled water to achieve the 0.2% and 0.02% solutions. One mL of each solution was mixed with 1 mL of a suspension of planktonic cells from an advanced exponential growth phase culture, to achieve the same final chitosan concentrations used in the biofilm assays.

### 3.4. Cell Recovery and Counting

For counting biofilm cells, coverslips were withdrawn with tweezers and dipped for 1 min in saline (0.9% w/v). The cells attached to the coverslip were removed by swabbing both coverslip faces and were dispersed into 1.5 mL peptone water. Tubes were then vigorously stirred in a vortex to break up aggregates. In the case of planktonic cells, suspensions were diluted for plating. For both biofilms and planktonic cells, 100 μL of all decimal dilutions (ranging from 0 to 5) were pour-plated on TSA (Oxoid). Colonies were counted after incubation at 37 °C/48 h, or 30 °C/48 h in the case of *Pseudomonas*. All assays were run in triplicate and 2 coverslips from each carousel, or 2 test tubes in the case of planktonic assays, were used for counting (in total, n = 6).

### 3.5. Statistical Analysis

Data were analyzed using Statgraphics Plus 5.0 software (Statistical Graphics Corporation, Rockville, Md., USA). ONE-way ANOVA analysis of variance and Duncan’s procedure for multiple mean comparisons were carried out to determine if treated and non-treated samples were significantly different at a 95.0% confidence level (p < 0.05).

### 3.6. Confocal Scanning Laser Microscopy (CSLM)

Biofilms were stained with the Film Tracer^TM^ LIVE/DEAD biofilm Viability kit (Invitrogen). This kit includes Syto^®^9, which stains all the cells and propidium iodide which only penetrates cells with injured membranes. Thus, green cells would correspond to cells with intact membranes (live), whereas red cells would have previously damaged membranes (dead). CSLM images were obtained using a TCS SP2 model (Leica Lasertechnik, Heidelberg, Germany). The stained biofilms were examined using a water immersion objective lens 63X. Three-dimensional projections of biofilm structure and extended section views were reconstructed from z-stacks using IMARIS^®^ software (Bitplane AG, Zúrich, Switzerland).

## 4. Conclusions

A log cell reduction of about 6 was achieved exposing *L. monocytogenes* biofilms to 1% native chitosan for 60 min at 20 °C. This is an excellent result for this species taking into account that their laboratory biofilms do not usually surpass a cell density of 10^6^ CFU cm^−2^. Also very satisfactory was the result of an 8 log reduction of biofilm cells of *B. cereus* obtained with 1% COS treatment. Effectiveness on the other species was lower but still useful for cases of not very developed biofilms. Very insufficient results on *S. aureus* were the poorest outcome of these attempts. Higher susceptibility of biofilm cells than planktonic cells (except with *S. aureus*) to 1% chitosan was an unexpected result, deserving further more detailed work.

## Figures and Tables

**Table 1:** Log cell reduction after 60 min immersion in 0.1% chitosan of mature biofilms of several *Listeria monocytogenes* strains (n = 6).

Strain	Serovar	Log reduction	SD
Scott A	4^b^	4.04^a^	0.10
INIA H66^a^	1/2^a^	3.02^b^	0.62
INIA H66^b^	1/2^c^	2.63^b^	0.28
INIA H63^a^	1/2^b^	1.47^d^	0.18
INIA CAL17^a^	4^b^	2.01^c^	0.08
F6861	4^b^	1.84^c^,^d^	0.08

a,b,c,d: values showing different letters are significantly different (p < 0.05).
